# Feedlot Cattle Antimicrobial Use Surveillance Network: A Canadian Journey

**DOI:** 10.3389/fvets.2020.596042

**Published:** 2020-11-20

**Authors:** Sherry J. Hannon, Stephanie A. Brault, Simon J. G. Otto, Paul S. Morley, Tim A. McAllister, Calvin W. Booker, Sheryl P. Gow

**Affiliations:** ^1^Feedlot Health Management Services Ltd., Okotoks, AB, Canada; ^2^Department of Clinical Sciences, College of Veterinary Medicine & Biological Sciences, Colorado, State University, Fort Collins, CO, United States; ^3^School of Public Health, University of Alberta, Edmonton, AB, Canada; ^4^Veterinary Education, Research, and Outreach Center, Texas A&M University and West Texas A&M University, Canyon, TX, United States; ^5^Agriculture and Agri-Food Canada, Lethbridge, AB, Canada; ^6^Canadian Integrated Program for Antimicrobial Resistance Surveillance, Public Health Agency of Canada, Saskatoon, SK, Canada

**Keywords:** feedlot cattle, antimicrobial use, surveillance, Canada, AMU

## Abstract

Antimicrobial drugs are important tools for maintaining human and animal health. Globally, antimicrobial use (AMU) in food-producing animals is under increasing scrutiny due to its potential to promote antimicrobial resistance (AMR). Historically, comprehensive Canadian data related to the types of antimicrobial drugs used, extent of use, common indicators of use and the demographics of the cattle populations receiving antimicrobial drugs have been limited, in part due to segmentation in the cattle industry and fragmentation of the drug distribution system. Appropriate AMU estimates are required to understand AMU practices, to interpret AMR levels and patterns, to meaningfully assess associated public health risks, and to inform stewardship activities. The Canadian beef cattle industry has a long history of collaboration in AMU and AMR research. Prior research projects identified both opportunities and challenges in the collection of AMU data. Cornerstone projects provided insight into the complexity of collecting AMU data in Canada's feedlot sector. This paper will discuss how the lessons learned from past work have contributed to the formation of a Canadian fed-cattle antimicrobial surveillance program that was initiated in 2019. This important surveillance program will allow feedlot cattle AMU to improve management decisions and support AMU best practices in the evolving Canadian AMR landscape.

## Introduction

Global concerns about the impacts of antimicrobial-resistant bacteria in public health have led to increased scrutiny of antimicrobial use (AMU) practices in food producing animals (including feedlot cattle) (1). The majority of Canadian feedlot production (78%) and cattle slaughter (76%) occur within western Canada (2) and in Alberta specifically. Historically, the Canadian beef industry has taken a proactive leadership role in antimicrobial stewardship and knowledge acquisition by funding high quality research related to AMU and antimicrobial resistance (AMR); see Table 1. Successful research collaborations have amassed a large body of AMU-related research (3–24). Herein, we discuss the Canadian feedlot cattle sector's journey to organize a national system for AMU data collection. Cornerstone projects that helped identify and fill knowledge gaps are described, but this paper is not a systematic review and selectively highlights peer-reviewed publications from publicly funded projects that incorporate or relate specifically to AMU data collection and use. This paper will review successes and challenges related to field implementation, data collection and summarization of results in these collaborative AMU surveillance efforts, as well as how the lessons learned from past work have contributed to the formation of the Canadian Fed-cattle Antimicrobial Surveillance Program [Gow et al., 2018, Table 1; (CanFASP)] that was initiated in 2019.

**Table 1:** Large-scale Canadian projects contributing to the body of work on AMR in feedlot cattle, with the important inclusion of AMU data as part of the research.

**Funding timeframe Data collection dates**	**Study title**	**In-text reference; Date of final report**	**Examples of AMU-related references**
1999–2004 Sep 1999 to Aug 2000	Investigation of AMR in bacteria isolated from beef cattle and potential transmission to humans^a^	Read et al., 2004	Unpublished
2003–2005 Mar 2004 to Dec 2004	Baseline prevalence of antimicrobial resistance foodborne and indicator bacteria in Alberta feedlots^b^	Van donkersgoed et al., 2006	(3)
2006–2010 Cattle placed Sep 2007 to Jan 2010	Development of a longitudinal AMR and AMU surveillance program for the feedlot sector in Canada^c^	Gow et al., 2012 PROSPECTIVE	(4–9)
2013–2018 Apr 2014 to Jan 2016	Surveillance of *E.coli*, enterococci, antimicrobial resistance and *Enterococcus* species distribution in beef operation-associated environments^d^	McAllister et al., ONEHEALTH	(10–13)
2015–2018 Cattle placed Nov 2008 to Oct 2012	Describe/understand collection of AMU data from a representative population of the Canadian feedlot sector using *i*FHM*S*^e^	Hannon et al., 2018, RETROSPECTIVE	(14, 15)
Surveillance program 2018 - present Jul 2019 - present	Surveillance of AMU and AMR in Canadian feedlot cattle (Canadian fed-cattle antimicrobial surveillance program)^f^	Gow et al., 2018, CanFASP Initiated in 2019	Unpublished

a *The Canada-Alberta Beef Industry Development Research Fund (#98AB272)*.

b *The Alberta Beef Industry Development Research Fund and Alberta Agriculture Research Institute (#2003L005R) and Alberta Beef Producers (#2004-01)*.

c *Advancing Canadian Agriculture and Agri-Food Program; the Alberta Beef Producers, (#0007-038RDB); the Canadian Cattlemen's Association, Canadian Beef Cattle Checkoff (Beef Cattle Research Council, #BCRC6.41) and the Surveillance Division, Center for Food-borne, Environmental Zoonotic Infectious Diseases, Public Health Agency of Canada, Canadian Integrated Program for Antimicrobial Resistance Surveillance*.

d *Canadian Beef Cattle Checkoff (Beef Cattle Research Council), Alberta Beef Producers and Agriculture and Agri-Food Canada, (FOS 10.13)*.

e *Canadian Beef Cattle Checkoff (Beef Cattle Research Council), Alberta Beef Producers and Agriculture and Agri-Food Canada, (administered under FOS 10.13)*.

f *Alberta Cattle Feeders; Canadian Agriculture Partnership: Alberta; Canadian Agriculture Partnership: Ontario; Canadian Beef Cattle Checkoff (Beef Cattle Research Council); Bayer Animal Health; Beef Farmers of Ontario; McDonalds Corporation; National Cattle Feeders Association, Public Health Agency of Canada/Canadian Integrated Program for Antimicrobial Resistance Surveillance; Saskatchewan Agriculture; Saskatchewan Cattlemen's Association; Vetoquinol*.

### Targeted Research

Lack of accurate data creates a void that may be filled with inaccurate estimates, theories and assumptions (25, 26). Initially, Canadian researchers collected baseline antimicrobial susceptibility data for bacteria found in cattle that may impact either human or animal health (3, 4, 17–20). When feasible, studies included isolates collected from human subjects to assess public health implications (4, 19, 20). One of the first large Canadian feedlot industry collaborative studies (Read et al., 2004, unpublished; Table 1), investigated the prevalence and AMR profiles of bacteria linked to human health impacts such as campylobacters, salmonellas, vancomycin-resistant *Enterococcus*, and methicillin-resistant *Staphylococcus aureus*. Generic *Escherichia coli* was also used as an indicator organism, and fecal and nasal samples were collected from cattle and feedlot workers to assess occupational hazards (Read et al., 2004, Table 1). As well, this project collected important antimicrobial susceptibility information for Canadian feedlot cattle producers on nasal bacteria including *Pasteurella multocida, Mannheimia haemolytica*, and *Haemophilus somnus* [now *Histophilus somni*]. These nasal bacteria are critical components of the bovine respiratory disease complex which is the most important disease syndrome in feedlot cattle production globally (27, 28).

Publicly funded and targeted research continued to evolve to understand AMR in light of changing feedlot cattle management and AMU practices, to identify trends over time, to improve Canadian AMU transparency, and to move ever closer to the establishment of a national feedlot cattle AMU surveillance program [Van donkersgoed et al., 2006, Table 1; (3, 16–18)]. A prospective cohort study on feedlot cattle in western Canada (Gow et al., 2012, Table 1; PROSPECTIVE) continued feedlot cattle AMU data collection and AMR profiling, and helped leverage support for important feedlot cattle/public health research on *Clostridium difficile* (19), MRSA (20), campylobacters (4), and *M. haemolytica* (5, 6). From 2013 to 2018, a large collaborative research project on *E.coli* and enterococci was conducted to provide information to producers and scientists about AMR, including macrolide (a commonly used antimicrobial in both human and animal health) use, based on a “One Health” approach. This project included samples from feedlot cattle, water (feedlot catch basin and surface), soil, and human clinical isolates, as well as feedlot AMU data [McAllister et al., Table 1, ONEHEALTH]. This study complemented other Canadian research on feedlot cattle AMU at that time (21–24); and a retrospective study mining AMU data from cattle placed in 36 western Canadian feedlots from 1-Nov 2008 to 31-Oct 2012 representing over 2.6 million animals from a proprietary and standardized data collection and storage system [*i*FHM*S*, Feedlot Health Management Service Ltd. (Feedlot Health), Okotoks, Alberta]. This project (Hannon et al., 2018, Table 1; RETROSPECTIVE), as well as the contributions from other Canadian researchers helped to identify data points meaningful for a national surveillance system, highlighted challenges in surveillance system development, and piloted data collection processes in preparation for future expansion in scope. These large collaborative projects, as well as other ongoing local, national and international initiatives, resulted in publications exploring metrics for understanding and interpreting feedlot cattle AMU data (7, 8, 14, 29, 30). These methodological papers were critical to developing indicators and evaluating metrics within the Canadian feedlot production system applicable to a future surveillance system.

### Canadian Policy Related to AMU and AMR

While targeted research continued, Canadian policy began to change based on emerging AMR in public health (Canada and worldwide) and the possibility that AMU in animals promotes resistance in humans (31, 32). In 2012, the National Beef Research Strategy (33) identified target research outcomes related to the incorporation of on-farm AMU and resistance data into ongoing national One Health surveillance programming. At that time, a standardized approach to feedlot cattle AMU data collection had not been adopted in Canada, although the PROSPECTIVE study had provided a basic framework which was further refined with the RETROSPECTIVE data and experience.

Over time, Health Canada began to change the Food and Drugs Regulations. The Canadian Council of Chief Veterinary Officers AMU in Animal Agriculture Committee completed an impact assessment for moving medically important antimicrobials (MIAs) to the prescription drug list using surveillance data from Canadian provinces (34), coupled to a review and assessment of AMU surveillance options for animals in Canada (35). Since 2018, mandatory reporting of antimicrobial drug (AMD) distribution for sale for animal use is required and all MIAs have been moved to the prescription drug list (36, 37). Regulation now prevents the importation of MIAs for own-use, requires that imported active pharmaceutical ingredients are only in approved forms from registered production facilities, and bans the use of MIAs for strictly growth promotion (38).

At a consultation event related to the 2016 National Beef Antimicrobial Research Strategy (39), informal discussions in support of large scale AMU data collection took place between the Canadian Integrated Program for Antimicrobial Resistance Surveillance (CIPARS) representatives, beef industry representatives and veterinary management groups. In the summer of 2018, with the recommendation of national and provincial cattle feeder and industry associations, an expert group was convened to work with CIPARS to draft a framework for sample and data collection in the feedlot sector related to AMU/AMR. The expert group consisted of the beef industry representatives, major veterinary practitioners involved in feedlot medicine across Canada, and AMU/AMR technical experts. The team developed a sentinel farm, sample collection, AMU surveillance, and veterinary AMD dispensing data collection frameworks; the CanFASP. Prior research conducted in Canada related to feedlot cattle and other livestock AMU/AMR, and the subsequent networks and partnerships that arose, were instrumental in the design and implementation of these frameworks.

## Lessons Learned and Recommendations

As per the Centers for Disease Control and Prevention, surveillance is “the ongoing, systematic, collection, analysis and interpretation of health-related data essential to the planning, implementation, and evaluation” of a system (40). As outlined in Teutsch and Churchill, there are a number of factors that contribute to the development of a successful surveillance system (41). These include clear objectives and case definitions, accurate and reliable data sources and collection instruments, appropriate field testing, development and testing of the analytic approaches, establishing useful and timely dissemination mechanisms, and looping back to ensure the analyses and interpretations are meaningful (41). Each of the past Canadian feedlot cattle industry research projects built on the “lessons learned” (see Table 2), from previous endeavors to formulate the current feedlot surveillance frameworks. The current systems are continually evaluated, re-evaluated and streamlined to improve usability, optimize resources, and troubleshoot barriers to smooth functioning. The following sections outline some areas identified in past research that were used to design the CanFASP, as well as recommendations from the knowledge gained. Methodologies in each of the targeted AMU and/or AMR research projects described in Table 1 differed based on individual project goals.

**Table 2:** Challenges and lessons learned from large-scale Canadian feedlot cattle AMU projects.

**Design**
Surveillance allows for emerging trends to be identified
Targeted research is critical prior to, and after surveillance system implementation to optimize data collection, summarization, reporting and dissemination
A specifically designed and dedicated system is required to efficiently collect and store individual-animal administered and in-feed AMU data at the national level
Piloting is critical to identify meaningful data points, without overburdening data suppliers
Surveillance design must be flexible so changes in feedlot production can be successfully managed
Surveillance should include pathogens of both public health and animal health importance to benefit the most stakeholders
The ability to compensate producers and veterinarians for their time and access to inventory or sites encourages ongoing participation
Sustainable, long term funding to support human and infrastructure resources and system maintenance is needed
**Sampling and data collection**
Schedule sampling so diagnostic infrastructure is not overwhelmed
Individual-animal administered AMU data access and compilation is straightforward because computerized systems have been specifically designed for this
In-feed AMU data access and collection and compilation can be time consuming because feed data collection systems have not been designed specifically for this – multiple indirect data sources often required to estimate in-feed AMU
Sample collection at standard commercial handling timepoints promotes compliance and improves data accuracy
Composite samples are comparable to individual animal samples for *E. coli* AMR
Stakeholders asked to supply/upload AMU data need to understand the data formats ahead of time so that administrative changes can be made to facilitate collection and compilation
Prescriptions, dispensing records and AMU data do not measure the same things
In-feed cohort exposure assessment (particularly in the last $\frac{2}{3}$ of feeding) is difficult due to present production practices (animal mixing and sorting)
Compilation of feedlot AMU data aggregated to the lot-level has been the most useful to date
**Metrics and indicators**
Standardization per 100,000 animals was important for appropriate AMU data interpretation
The number of animal daily doses has been the most useful metric to date for Canadian feedlot systems
Average weight of heifers and steers (~360 kg) was the best weight estimate to use for approximating actual AMU in the Canadian feedlot system
Subset data for individual-animal administered AMDs may be representative of census data at feedlots, depending on the goal/objectives
Summarizing AMDs of low human health importance separately from those of moderate/high importance seems most useful and least biased compared to other countries
**Dissemination**
Site-specific as well as aggregated result reports provide value to producers for ongoing participation

### Design Recommendations

#### Experienced Teams to Implement and Manage the System

For the CanFASP, we identified five key veterinary management groups/consulting practices that provide animal health services and production advice to feedlot producers representing a large proportion of Canadian fed cattle. These veterinary management groups often have proprietary, standardized, data collection and data management software systems (e.g., *i*FHM*S*), or use specific commercial software programs to collect individual-animal data at each chute (processing barn, hospital) within a client's feedlot. Feedlot personnel trained in the use of each feedlot's computer system are required for accurate real-time data collection at the time of animal handling. Veterinarians are critical for providing prescription information and summarizing the AMD administration program at each site as they understand why and how the AMDs are used. Veterinary management group team members often oversee animal sample collection and shipping, as well as the compiling of data for surveillance system administrators. Laboratory personnel manage standardized laboratory analysis of animal samples and communicate laboratory results to the surveillance system administrators. Surveillance system administrators must coordinate all moving parts of the system including sample submissions, laboratory analyses, standardized data uploads, summarization and interpretation of findings, timely dissemination of results to stakeholders, random troubleshooting/enquiries, and assisting with ongoing sourcing of funds to support the network. All the above teams are part of the necessary infrastructure for the appropriate and accurate collection and assembly of the surveillance data. Strong communication between these teams is essential for optimum functioning of the surveillance system.

#### Understanding and Prioritizing Surveillance Objectives

To collect the most meaningful data for understanding cattle AMU on a national scale, the objectives and anticipated outcomes of the surveillance initiative must be clear and specific (41). To do this, it is critical that the system is designed with input from those knowledgeable about all aspects of the production system and with input from all key stakeholders. All data come at a cost (i.e., to veterinarians, producers, and the surveillance system), and the cost-benefit of each piece of data must be considered. Understanding the specific objectives and identifying the data points necessary to achieve these, along with the ability to revisit/redefine objectives and data points over time is critical (41). A great deal of effort was spent prioritizing and specifying objectives and outcomes for the CanFASP, including rounds of consultation and discussion within the expert group to ensure that the program scope was broad enough to be useful to the majority of stakeholders, while appropriately focused to be practical and cost effective for implementation. The objectives of the CanFASP are presented in Supplementary Table 1.

To address challenges around the cost-benefit and logistical issues of implementing a national surveillance project and to ensure that the program was relevant to stakeholders, the Canadian approach developed inclusion/exclusion criteria that aligned with how the majority of feedlot cattle in Canada are raised (Supplementary Table 2). Based on the inclusion/exclusion criteria, each of the participating five major feedlot veterinary management groups provided an anonymized list of their practices feedlots that met the inclusion criteria. The feedlot bunk capacity was also provided for each feedlot. Feedlots were randomly selected from a list of those eligible, after stratification by feedlot size (one-time bunk capacity of 1,000–5,000 cattle, 5,001–10,000 cattle, 10,001–20,000 cattle and >20,001 cattle) and veterinary management group. The number of cattle required within each size stratum was proportional to its contribution to the Canadian fed cattle population (42). The targeted number of animals required for the entire project was calculated using the same approach as described in Timmerman et al. (43) and based on treatment incidence numbers from the RETROSPECTIVE study. This approach was designed to be representative of the Canadian fed-cattle sector and to allow the program to evolve into a smooth streamlined surveillance system without overloading available human (veterinarians, producers, administrators) and financial resources.

#### Case Definitions

Specific and established case definitions are required to standardize data across provinces, feedlots and veterinary management groups compiling the data. Within each research project, and then later within the CanFASP, case definitions, response formats and standardized categories for responses were defined and agreed to *a priori*, to facilitate accurate data collection and summarization. Although the CanFASP definitions will be regularly revisited to ensure relevancy, the intent is to minimize changes in scope to allow data comparability over time.

#### Identifying the Best Stakeholder(s) to Supply the Surveillance Data

The CanFASP was designed to have veterinarians work with the feedlots to collect data, as do the other CIPARS farm AMU programs. CIPARS has traditionally worked with veterinarians because of their comprehensive understanding of the AMU on their client farms (improving data quality), existing veterinary-client-patient relationships, and their ability to ensure confidentiality. While producers have access to individual animal or group data, veterinarians are responsible for providing AMU oversight. If required, veterinarians could be compelled (through professional and/or government regulation) to report AMU as part of maintaining licensure, whereas there are no similar incentives to encourage producers to disclose AMU data. Compensation for the extensive time and effort currently needed to collect and report AMU information is important as these data are often sought for public good. If producers were the main target for data collection, much thought and planning would be required to keep them engaged, maintain data quality, demonstrate value, and compensate them for their time.

#### Confidentiality, Informed Consent, and Privacy

Success of a surveillance system requires protection of confidentiality and privacy. The approach to confidentiality used for the CanFASP is consistent with previous Canadian research projects including the PROSPECTIVE and RETROSPECTIVE studies, and other existing CIPARS farm programs. All data are coded and personal identifiers (e.g., feedlot and producer names, addresses etc.) are not provided to the CIPARS system administrators. The codes reside with the veterinary management group and are not available to external collaborators using the data. To protect privacy, data are publicly reported in an aggregated format and not reported by veterinarian or by veterinary clinic. This approach ensures that no attempt can be made to identify or directly contact participating producers. Additionally, informed consent is obtained, reviewed and signed annually by the enrolled producer and their coordinating veterinarian. This consent details the project, external collaborators, data required, reporting mechanisms and methods to protect privacy and confidentiality.

#### Specific Design for Data Collection

Challenges in data acquisition and organization arise when surveillance is not the intended purpose of the data (41). In the Canadian feedlot cattle industry, individual animal data recording systems are designed to be a version of computerized medical records and include direct recording of all individual-administered product use including AMDs. Summarizing these data is relatively straightforward because the collection/storage systems are designed for AMU data input and retrieval. However, in-feed AMU data summarization is not as straightforward because current feed-related data collection systems are designed to collect feed data not product data such as AMDs. For successful, accurate and efficient data acquisition, collectors must be able to access and assemble clearly defined/required pieces of information prospectively and easily. This does not mean that all raw data must come from the same data collection system(s), but they must be easily accessible. Furthermore, a standardized AMU storage system with mandatory fields and controlled data formats must exist for compilation, storage and timely summarization of data. The challenge is then to identify who bears the associated cost and responsibility to create, support and maintain the required infrastructure.

#### Field Testing Data Collection and Analytic Approach

Much of the knowledge related to the implementation of the CanFASP came from lessons learned from previous research, as well as from piloting of the surveillance system in 2019. Flexibility in the first year allowed feedback and discussion related to whether objectives, data collection procedures, laboratory analyses, results summarization and interpretation, and dissemination of information to stakeholders were successfully achieved.

Computer systems for individual-animal administered AMU (e.g., parenteral or oral bolus data) had been developed specifically for animal health and treatment tracking long before they were used for AMU surveillance efforts. While not developed for surveillance purposes, these systems are useful for obtaining accurate and comprehensive individual animal data.

Assimilating in-feed data is much more challenging, and combined approaches to in-feed data collection have been used historically and in the CanFASP. Prior to 2018, in-feed MIAs in the published Canadian Food Inspection Agency's Medicated Ingredients Brochure did not require a medicated feed prescription for on-label use, and, depending on the AMD, there was often no withdrawal time required prior to slaughter. Feed-related computer systems are not designed to specifically collect product use data including AMU, and as a result, these data are not directly recorded into these systems. Therefore, site-specific in-feed AMU data must be compiled from a variety of indirect sources depending on site-specific management and record keeping practices. These include veterinary AMD feed prescriptions, electronic or hard copy daily feed delivery records compiled and stored by the feedlot as part of daily operations, feeding standard operating procedures, and/or ration composition records. In commercial production systems, cattle are fed as a group within a pen rather than individually, so it is necessary to know which animals are in which pens to correlate pen feeding records to specific animal AMU. Depending on the AMD and its use, in-feed AMD dosages could be recorded as g/animal/day or as the mg/kg diet dry matter, and calculations are then performed to generate the total grams of active ingredient delivered/animal/day based on dry matter intake, the number of animals exposed and the duration of the exposure. This approach is much more complicated than directly inputting individual-animal administered AMU data into databases (15).

While in-feed AMU occurs under the direction of a licensed veterinarian with a valid veterinary-client-patient relationship, there are many rations fed each day at a single site, and each one may have a different AMD inclusion level based on the stage of the production cycle, ingredients required in each diet, and the overall management goals. Disease risk may change during the production cycle and the AMD used must therefore change as well. Even though the AMD inclusion levels in the rations may change to account for factors such as intake levels or dry matter content changes, ration names are often kept the same to facilitate daily feedlot operations. Ration reference tables with date ranges specifying AMD inclusions during each range are not commonly maintained in most systems. This makes it challenging to identify the exact daily AMD concentration for each ration, fed to each pen. While veterinary medicated feed prescriptions are often available, they do not necessarily match the amounts dispensed by feed mills with partially filled prescriptions, or the amounts used by producers as they only use what is needed. Understanding the potential differences in these three sources of AMU data is critical. It continues to be a priority to find ways to mine in-feed AMU data from feeding software and administrative databases more accurately and efficiently. For the CanFASP, veterinarians could work with producers prior to data collection to streamline naming conventions to more easily identify the antimicrobial content of the rations, to develop forms for recording AMU-related data points that were not easily captured previously, and/or to create custom reports in software systems to facilitate data extraction.

In feedlot management systems, a production lot is an administrative entity used to track financial costs and revenues associated with specific groups of cattle. In general, all cattle are assigned to a production lot at the time of feedlot entry, and once animals in the production lot have exited the feedlot, a “lot closeout” is created to summarize the main lot-level indices. Animals within a production lot may be procured all at once or in groups over time, depending on the management practices or business model of the feedlot. Similarly, cattle within the same production lot may be marketed all at once or in groups over time, due to differences in growth rates and other cattle management logistics (e.g., beta-agonist feeding). At the start of the PROSPECTIVE study, cattle tended to stay with the same pen-mates over the entire feeding period, and the study was designed to evaluate pen member exposures. By the end of the study, changing industry practices often resulted in sorting/mixing of cattle from different pens during the feeding period. Animal sorting/mixing makes it extremely challenging to track pen-level cohort exposure and, as a result, production lot-level summarization has been employed in subsequent studies.

There is international pressure to benchmark AMU or to have census data (44). However, when designing a surveillance system, data accuracy and usability must be balanced against practical and logistical considerations and the cost of data collection. Preliminary analysis of census data provided in the RETROSPECTIVE study and supported by the PROSPECTIVE study found that individual-animal feedlot cattle AMU collected from a targeted subset of animals within a veterinary practice could be representative of that feedlot (unpublished data). However, subset data may need to be interpreted carefully by policy makers and stakeholders depending on the specific generalizations to be made. As sampling animal subsets rather than collecting census data can be much more cost-efficient and potentially provide more consistent results (given that feedlot-to-feedlot differences may have less impact), future research in this area should assess the validity of this approach.

#### Adaptability Over Time

Feedlot cattle AMU changes over time for a number of reasons, including revisions to industry-led animal health protocols and management practices, clinical field trial research results, regulatory changes, and international guidance. For a national AMU surveillance system to be successful, it must be flexible so as to adapt to the dynamic nature of the industry, while still allowing for comparability over time. It is expected that veterinary management groups will continue to optimize data collection through software development, targeted data mining strategies, and *a priori* collaborations with feedlot management to streamline AMU data collection critical control points such as ration naming, composition recording, and access to feed data. As well, development continues on the integrated database that receives, stores and maintains the CanFASP AMU surveillance data to improve system efficiency and the speed at which analyses and results can be compiled and distributed to industry stakeholders, veterinary management groups and producers. The CanFASP has funding for three years from 12 different sources (Table 1), including the feedlot industry, pharma, retail, federal, and provincial governments, with extensive voluntary time contributions from government, academia, veterinary practice, feedlot producer and industry stakeholders. However, without dedicated funding and human resource allocation, continuation, maintenance, and upgrading of the system will be a challenge.

### Data Recommendations

#### Raw Data Collection Systems

Regardless of the computerized data collection system used at each feedlot, it is key that: (1) feedlot team members are appropriately trained and engaged to capture all relevant data points in real-time, and (2) the computerized data collection system is sufficiently user-friendly to ensure data entered at the feedlot are as accurate and complete as possible. Once data have been entered, veterinarians or veterinary management group team members compile data for upload to surveillance system administrators. It is critical that the database into which the raw data are entered can be specifically mined to allow relevant data points to be searched, identified and efficiently compiled into a standardized format for reporting.

Individual-animal data collected must provide sufficient detail for required AMU calculations. Identifying in-feed antimicrobials is relatively simple (with only oxytetracycline, chlortetracycline, and tylosin phosphate commonly administered in feedlot production). However, for group-level in-feed AMU data, accessing accurate data is complicated and time consuming as described above. See Supplementary Table 3 for data points currently collected in the CanFASP for individual-animal administered and in-feed AMDs. Over the years, some feedlot producers have moved from having commercial feed mills dilute concentrated AMDs into carrier supplements that are mixed into each load of feed, to using on-site automated micro-dispensing units to directly add small amounts of concentrated AMDs as each load of feed is mixed. Data recorded by these micro-dispensing units provide a direct measurement of in-feed AMU, but the micro-dispensing unit data needs to be cross-linked to each individual feed load delivered to each of the pen(s), and to the animals in the pen(s) when that load is fed. Currently, most Canadian feedlots lack a system to link these important data.

#### Surveillance Data Collection Systems

Once compiled, data should be uploaded into a centralized surveillance program database in a formatted and standardized manner so that the administration team members can easily oversee the incorporation of uploaded data. There are several ways to ensure standardization, including enforcing case definitions, using protected software to ensure data entry mistakes are not made, and promoting excellent communication between surveillance system administrators and those compiling AMU data during the upload phase.

#### Indicators

Currently, the international community lacks consensus as to the most appropriate indicator to describe AMU, which depends on the intended objectives of the surveillance system. In the RETROSPECTIVE study, census data that included detailed individual animal parenteral data and calculated in-feed data allowed for accurate calculation of AMU (14, 15). This level of resolution for AMU in animal production systems is often unavailable. The RETROSPECTIVE dataset encompassed almost a quarter of the fed cattle in Canada at the time of the study, making it an important contribution toward informing policy regarding the development of AMU surveillance systems suited to the Canadian beef industry.

The RETROSPECTIVE study demonstrated that vast differences in interpretation of AMU are possible depending on the indicator selected (14). For example, because the physical amount of active ingredient required for some macrolides is much lower than for some tetracyclines, when reporting individual-animal AMU in grams of AMD used, the macrolide use examined appeared to be ~6% of the tetracycline use examined (14). However, when reporting number of animal daily doses (which accounts for body weight and number of animals), the macrolide use examined was ~62% of the tetracycline use examined (14). Calculating these indicators highlights the importance of understanding which indicator is the best choice for each surveillance objective. Often more than one indicator may be necessary to appropriately describe data. Ongoing surveillance in Canada requires the identification of those indicators that are the most appropriate for surveillance system objectives and that facilitate accurate understanding by stakeholders. However, indicators may require further refinement for inclusion in national databases such as those used by CIPARS or for international comparisons.

For many AMU-related calculations, there is a requirement for standardized animal weights. The European Surveillance of Veterinary Antimicrobial Consumption (ESVAC) has specified standardized weights for many species including beef cattle. Based on actual weight at treatment for parenteral AMD administration in the RETROSPECTIVE study, it was found that an average heifer and steer weighed ~360 kg, and as a result this value was used for approximating AMU when a standardized weight was required, such as for the calculation of the population correction unit (PCU) (14, 45). Using the ESVAC standardized weights for heifers (200 kg) and bulls/bullocks (425 kg), PCU calculation overestimated and underestimated AMU, respectively. As a result, for each country, standardized weights calculated from actual data are preferable to a common global standardized weight (14, 45) for representing actual AMU within a country. While a global standardized weight may be desirable for international comparisons, the AMD dose animals receive is often based on body weights, which differ among production systems. Therefore, if a country has heavier animals it will result in greater AMU. The total milligrams used will be a reflection of the size and number of animals, and results may be construed if non-representative weights are used. One group in the US is using kg of live weight sold as a denominator (46). Slaughter weight provides an opportunity to describe the amount of AMD required to reach slaughter weight or the amount of AMD to produce a kg of beef; metrics that are easily understandable by both producers and the public.

Standardization per 100,000 animals or 100,000 animal days-at-risk is also an important way of ensuring appropriate AMU interpretation when denominator numbers change over time (47). For in-feed antimicrobials, one factor that may contribute to AMU differences over time is animal days on feed. Factors such as seasonal temperatures and management, placement weights, shipment weights, and rates of gain all contribute to the number of days animals stay in the feedlot. CIPARS farm programs, including the one for feedlot cattle, currently include indicators for standardization by animal-time.

### Reporting Recommendations

#### Stakeholder Communication

There is a wide and diverse group of stakeholders that seek data about feedlot cattle AMU. These include but are not limited to government, academia, policy makers, pharmaceutical companies, commodity organizations, veterinarians, producers, and the public (48). The best approach to convey key messages to stakeholders for both AMU and AMR data has not yet been clearly identified. The AMU data are complicated to explain, and often require an in-depth understanding of the topic to comprehend important nuances in calculations and indicators. Without a way to share results so that the appropriate stakeholders can meaningfully act on the findings, the power and purpose of the surveillance system is diminished. It is unlikely that there is a single approach to reporting AMU data that will be useful to all stakeholders that desire these data. For the CanFASP, an annual aggregated summary report will be prepared, as well as reports to individual feedlots comparing their data to national indices. Additional work is required to explore how to communicate the results more effectively to all stakeholders, and to ensure that the goals of the system are successfully met. CIPARS is working toward an interactive data display that allows users to customize data displays to their needs. However, the human and financial resources required to generate annual reports or more real-time, interactive displays are considerable, and must be considered in the planning of any surveillance system (49, 50).

#### Local and Global Comparability for Total AMU

When designing a surveillance system, it is important to consider how the information will be used and by whom. Of all in-feed AMU (medically important and non-medically important AMDs as classified by Health Canada) in the RETROSPECTIVE study, ionophores represented >89% of administration (number of animal daily doses) with monensin use in >99.9% of cases. Ionophores are used primarily for coccidia control, but also to improve feed efficiency and prevent digestive disturbances. As a class of antimicrobials currently designated as low importance to human health, their inclusion greatly inflates AMU estimates and detracts from understanding AMU trends in MIAs. As some countries do not classify ionophores as AMDs, there is potential for wide variations in reported total AMU that is simply a result of ionophore inclusion/exclusion in reported results. The current situation in Canada is that ionophores and chemical coccidiostats are reported separately from MIAs (51).

### Other Recommendations

#### Continuing Research

In the Canadian beef industry, large pen commercial field trials are used to compare AMDs, animal health, and feeding programs, and often result in changes to management protocols and AMDs recommended to producers (standardized consulting protocols). Research in the commercial production setting plays a critical role in changing/reducing the amount of AMU over time. For example, large scale field trials identified that chlortetracycline use for histophilosis control could be significantly reduced in animals receiving metaphylactic tulathromycin (Feedlot Health; unpublished data). This research was driven by the need to improve animal health and welfare (reduce morbidity and mortality) and maintain feedlot production sustainability. These results translated into AMU protocol changes that highlight the importance of research as a tool to support antimicrobial stewardship.

Research based on infrastructure from existing surveillance systems can provide critical information to drive interventions and change. Results from the CanFASP and other national surveillance systems may generate new research hypotheses. Opportunities to conduct research through existing surveillance infrastructure will be evaluated on a case-by-case basis. Support will be prioritized to projects that can use previously collected samples or data, or which can be conducted within the resource allocation of the existing system. For example, a current initiative funded by Genome Canada will use some of the comprehensive data from the RETROSPECTIVE study combined with genomic data from novel platforms to develop, inform and strengthen the accuracy of predictive animal health disease transmission models (52, 53). It is hoped that the CanFASP and other Canadian feedlot cattle research can continue to support and progress initiatives such as this.

## Discussion

Data from previous targeted Canadian AMU-related research provide a solid AMU data collection foundation for ongoing AMU surveillance in the feedlot industry. CIPARS has shown that nationally and provincially representative commodity specific AMR and AMU data may be used to describe trends over time and between regions, investigate associations with relevant risk factors, educate stakeholders, inform policy development, provide baselines for international trade and AMR monitoring groups, and support antimicrobial stewardship (51, 54). Similar benefits are hoped to result from the ongoing surveillance of feedlot cattle AMU data in Canada based on the CanFASP.

Based on our experience (7, 9, 14, 15), the compilation, summarization and interpretation of individual-animal and in-feed AMU data is a considerable undertaking. The number of individual-animal AMDs can be high compared to in-feed, but because of purposely designed and often standardized raw AMU data collection tools, it is often more practical to compile and easier to summarize than in-feed data (15). Compiling census feedlot cattle AMU data is possible (15), however, without standardized electronic data collection methods/tools designed to capture in-feed AMU data and the uptake of these tools by feedlot operators/owners, the collection and summarization of these data on the scale required for robust, representative and useful data over time is not resource sustainable in our experience. The CanFASP launched in 2019 builds on previous research and continues historically strong collaborative efforts between the Canadian beef industry, academic and government stakeholders in the area of feedlot cattle AMU/AMR research. The ongoing development of this system has the potential to benefit many agricultural commodity groups (locally, nationally and internationally) as they all work to improve AMU oversight, to maintain and improve animal welfare, to promote antimicrobial stewardship and to support public health.

The development of a feedlot cattle AMU data surveillance system in Canada is intended to remove gaps in knowledge, to provide practical data collection recommendations, to provide transparency on feedlot production practices, to improve understanding of AMU in feedlot cattle and to support beef production, international trade, and access to AMDs for Canadian beef producers.

## Author Contributions

SG was the primary investigator of the PROSPECTIVE study and is the development lead/coordinator of the CanFASP. TM was the primary investigator of the ONEHEALTH study. SH was the primary investigator of the RETROSPECTIVE study. SB was instrumental in data analysis of the RETROSPECTIVE study. SH, SG, and SO drafted the manuscript. All authors made significant contributions to one or more projects described in this manuscript, participated as members of the expert consultation group related to the development of the CanFASP, and participated in manuscript revision.

## Conflict of Interest

CB is part owner and managing partner of Feedlot Health Management Services Ltd. and Southern Alberta Veterinary Services. SH is an employee at Feedlot Health Management Services Ltd., Okotoks, Alberta, Canada. Feedlot Health is a private company that provides expert consultation regarding production and management of feedlot cattle and calf grower calves, including developing veterinary protocols to support animal health. They also conduct in-house and contract research related to dairy calf grower and feedlot production. The remaining authors declare that the research was conducted in the absence of any commercial or financial relationships that could be construed as a potential conflict of interest.

## Abbreviations

AMD: antimicrobial drug
AMU: antimicrobial use
AMR: antimicrobial resistance
BCRC: Beef Cattle Research Council
CanFASP: Canadian Fed-cattle Antimicrobial Surveillance Program
CAHSS: Canadian Animal Health Surveillance System
CCAR: Canadian Committee on Antibiotic Resistance
CCVO: Canadian Council of Chief Veterinary Officers
CDC: Centers for Disease Control and Prevention
CIPARS: Canadian Integrated Program for Antimicrobial Resistance Surveillance
EMA: European Medicines Agency
ESVAC: European Surveillance of Veterinary Antimicrobial Consumption
GOC: Government of Canada
HHS: TAGGS: US Department of Health and Human Services Tracking Accountability in Government Grants System
MIA: Medically important antimicrobial (as per Health Canada)
PCU: Population correction unit
PHAC: Public Health Agency of Canada
USDA, NAHMS, United States Department of Agriculture: National Animal Health Monitoring System.
