# Supplementary material for: Feedlot Cattle Antimicrobial Use Surveillance Network: A Canadian Journey
Source: Front Vet Sci. 2020 Nov 20;7:596042. doi: 10.3389/fvets.2020.596042 (PMC7714776; doi:10.3389/fvets.2020.596042)
Supplement: Supplementary file 1 [file Data_Sheet_1.docx]

**Supplementary Material**

**Supplementary Table 1** Objectives of the Canadian Fed-cattle Antimicrobial Surveillance Program

| **Framework Development Objectives:** |
| --- |
| - Establish the infrastructure and resourcing requirements to support a sustainable national AMU and AMR surveillance program in the feedlot sector. - Be able to collect representative qualitative and quantitative data on AMU and AMR at the national and provincial/regional levels. Data will be summarized by region when there are a limited number of feedlots in a province and there is a risk of identifying participants based on feedlot capacity, or when there are expressed concerns that the full range of feedlot practices in that province are not being captured. |
| **Feedlot Surveillance Objectives:** |
| - Provide representative estimates of AMU and AMR in the Canadian finishing feedlot sector nationally and either provincially or regionally. Data will be summarized by region when there are a limited number of feedlots in a province and there is a risk of identifying participants based on feedlot capacity, or when there are expressed concerns that the full range of feedlot practices in that province are not being captured. - Provide a unified approach to monitor trends over time in AMU and AMR in the Canadian finishing feedlot sector nationally and either provincially or regionally. - Based on emerging trends in AMR, investigate associations between AMU and AMR periodically on a targeted basis. - Provide collated industry data for the assessment of the potential public and animal health risks of antimicrobials used in the Canadian finishing feedlot sector. |

**Supplementary Table 2** Inclusion exclusion criteria of the Canadian Fed-cattle Antimicrobial Surveillance Program

| **Inclusion criteria:** Participating veterinarians will identify and enroll clients that meet the following criteria**:** |
| --- |
| - Feedlots must be engaged in the finishing phase of cattle production; cattle not in the finishing phase may also be on site and included in the data collection, but at least some of the cattle on site must be sent directly from the feedlot to slaughter. - Participating feedlots must have a valid veterinary client patient relationship with the veterinarian enrolling the feedlot. - Each participating feedlot site must have a one-time capacity of > 1000 animals. |
| **Exclusion criteria**: Participating veterinarians will identify and ***not enroll*** clients that fall into the following categories: |
| - Feedlots that do not finish cattle to market weight. - Any feedlots that have a one-time production capacity of < 1000 animals. - Feedlots identified by the supervising veterinary clinic as not able to supply the minimum required data. |

**Supplementary Table 3** Raw data points collected as part of the Canadian Fed-cattle Antimicrobial Surveillance Program

| **Production lot** | **Feed Regimen** | **Injectable Regimen** | **Bolus Regimen** |
| --- | --- | --- | --- |
| Veterinary Code | Product name | Product name | Product name |
| Feedlot Code | Active ingredient | Active ingredient | Active ingredient |
| Production lot code | Antimicrobial class | Antimicrobial class | Antimicrobial class |
| # animals arriving | Route of administration | Route of administration | Route of administration |
| Average arrival date | Number of days fed | Product Concentration mg/ml | Bolus size mg |
| Average arrival weight* | Dose in g of active ingredient/ tonne of feed  OR  Dose in mg of active ingredient/kg body weight/day  OR  Dose in mg of active ingredient/animal/day | Dose mg/kg body weight  OR  Penicillins IU/ml  OR  Penicillins IU/kg body weight | Product concentration mg/bolus |
| Calf or yearling |  |  | Amount per body weight |
| Sex |  |  | Average # days administered |
| Origin (auction market, ranch direct, backgrounded) | Reason for use (liver abscess and coccidiosis control, histophilosis treatment or control) | Reason for use (metaphylaxis or treatment) | Reason for use  (metaphylaxis or treatment) |
| Risk of developing BRD | Condition for AMU | Condition for AMU | Condition for AMU |
| # animals slaughtered | Total number of times applied  OR  Total g of active ingredient used in the production lot | Total # of exposures  OR  Total g of active ingredient used in the production lot | Total # of exposures  OR  Total g of active ingredient used in the production lot |
| # animals that died | Total delivery of feed to lot (dry matter) |  |  |
| Average ship date |  |  |  |
| Average weight at exit* |  |  |  |
| Quarter of close out |  |  |  |

* Weight units
